# Estrogen Receptor α Is Crucial in Zearalenone-Induced Invasion and Migration of Prostate Cancer Cells

**DOI:** 10.3390/toxins10030098

**Published:** 2018-02-28

**Authors:** Karolina Kowalska, Dominika Ewa Habrowska-Górczyńska, Kinga Anna Urbanek, Kamila Domińska, Agnieszka Wanda Piastowska-Ciesielska

**Affiliations:** 1 Laboratory of Cell Cultures and Genomic Analysis, Department of Comparative Endocrinology, Faculty of Biomedical Sciences and Postgraduate Education, Medical University of Lodz, Zeligowskiego 7/9, 90-752 Lodz, Poland; klakus@op.pl (K.K.); dominika.habrowska@umed.lodz.pl (D.E.H.-G.); urbanek.kinga@gmail.com (K.A.U.); 2 Department of Comparative Endocrinology, Faculty of Biomedical Sciences and Postgraduate Education, Medical University of Lodz, Zeligowskiego 7/9, 90-752, Lodz, Poland; kamila.dominska@umed.lodz.pl

**Keywords:** zearalenone, mycotoxins, estrogen receptors

## Abstract

Zearalenone (ZEA), a mycotoxin produced in the genus *Fusarium*, binds to estrogen receptors (ER) and is therefore regarded as an endocrine disruptor. ZEA has also been found to modulate the proliferation and apoptosis of prostate cancer cells in a dose-dependent manner. This study evaluates whether the effect of a low dose of ZEA (0.1 and 0.001 nM) on the invasion and migration of prostate cancer cell line PC3 is associated with ERs expression. The invasion and migration was evaluated by modified Boyden chamber assay, scratch assay, gelatin zymography, Real Time qPCR (RTqPCR) and Western blot. The involvement of ERs was evaluated with the selective ER antagonists: estrogen receptor α (ERα) antagonist 1,3-*bis* (4-hydroxyphenyl)-4-methyl-5-[4-(2-piperidinylethoxy) phenol]-1H-pyrazole dihydrochloride (MPP) and estrogen receptor β (ERβ) antagonist 4-[2–phenyl-5,7–*bis* (trifluoromethyl) pyrazolo [1,5-a]-pyrimidin-3-yl] phenol (PHTPP). ZEA was found to modulate cell motility dependent on estrogen receptors, particularly ERα. Increased cell migration and invasion were associated with increased MMP-2 and MMP-9 activity as well as the up-regulation of the EMT-associated genes vimentin (*VIM*), zinc finger E-box-binding homeobox 1/2 (*ZEB1/2*) and transforming growth factor β 1 (*TGFβ1*). In conclusion, ZEA might modulate the invasiveness of prostate cancer cells dependently on ERα expression.

## 1. Introduction

Mycotoxin contamination is a global problem, concerning both developing and developed countries [1]. Mycotoxins, products of fungal metabolism, are present in human and animal food products and are stable under high pressure and temperature conditions [2]. Zearalenone (ZEA), chemically described as 6-(10-hydroxy-6-oxo-trans-1-undecenyl)-β-resocyclic acid lactone, is a secondary metabolite synthesized in the genus *Fusarium*, mainly *F. graminearum* and *F. cuolmorum* [3,4]. ZEA contaminates cereals before harvesting and is present in lower extend in meat, milk, wine, beer, dried fruit and spices [4,5] and might accumulates in the body [6]. The main harmful effect of ZEA derives from estrogenic activity, facilitated by its structural similarity to naturally-occurring estrogens and binding affinity to the estrogen receptors (ERs): ERα and ERβ [3]. ZEA is considered to act as an endocrine disruptor due to the fact that it is able to modulate the production of progesterone, estradiol, testosterone and cortisol [7], as well as hormone production and fertility and cause premature birth in domestic animals [3]. It is also well documented that ZEA modulates the process of cancerogenesis by influencing the process of apoptosis, ROS generation, the action of oxidative enzymes, DNA adduct formation and fragmentation [8,9]. It has been suggested that ZEA influences the incidence of breast cancer [10] and esophageal cancer [11]; it has also been observed to have a dose-dependent effect on prostate cancer (PCa) [12].

Motility is a key part of cell development and takes place both in physiological as well as pathological processes: embryogenesis, wound healing, tissue regeneration and cancer formation [13]. During the process of carcinogenesis, cell migration plays a central role in the metastasis and invasion of cancer cells [14]. The process of cell invasion is also associated with the epithelial- mesenchymal transition (EMT), resulting in the transformation of the cells: a process mainly associated with the expression of EMT markers and induction of cancer cell invasiveness [15]. Changes in the expression of transcription factors like Zinc finger E-box-binding homeobox 1/2 (ZEB1/2), Zinc finger protein SNAIL1 (SNAIL), Twist-related protein 1 (TWIST) or vimentin (VIM) might act as prognostic factors in the process of carcinogenesis [16]. Interestingly, a body of evidence indicates that ERs regulate the process of EMT through the expression of Transforming Growth Factor beta 1 (TGFβ1), E-cadherin and Hypoxia-Inducible Factor 1-alpha (HIF-1α) [17].

It has been found that ZEA might induce both apoptosis and proliferation in prostate cancer cells in a concentration-dependent manner [12]. The present study evaluates the effect of ZEA on the cell migration and invasiveness of the prostate adenocarcinoma cell line PC3, at two doses believed to have a stimulatory effect on PCa cells. It also determines whether the observed effect is associated with the expression of ERs. It uses the highly-specific ERα and ERβ antagonists 1,3-*bis* (4-hydroxyphenyl)-4-methyl-5-[4-(2-piperidinylethoxy) phenol]-1H-pyrazole dihydrochloride (MPP) and 4-[2–phenyl-5,7–*bis* (trifluoromethyl) pyrazolo [1,5-a]-pyrimidin-3-yl] phenol (PHTPP) to evaluate whether ERα or ERβ influence the observed effect of ZEA on PC3 cells.

## 2. Results

### 2.1. ERα is Responsible for ZEA- Induced PC3 Cell Invasion

The ICC was conducted to evaluate the expression of ERα and ERβ in PC3 cells before and during the experiment. Both receptors were present in PC3 cells in controls (Cnt) and ZEA with or without ERs inhibitors treated cells. Interestingly, 0.001 nM ZEA caused visible translocation of ERα to nuclei, indicating its activation, nevertheless this statement needs conformation in other studies.

**Figure 1:**
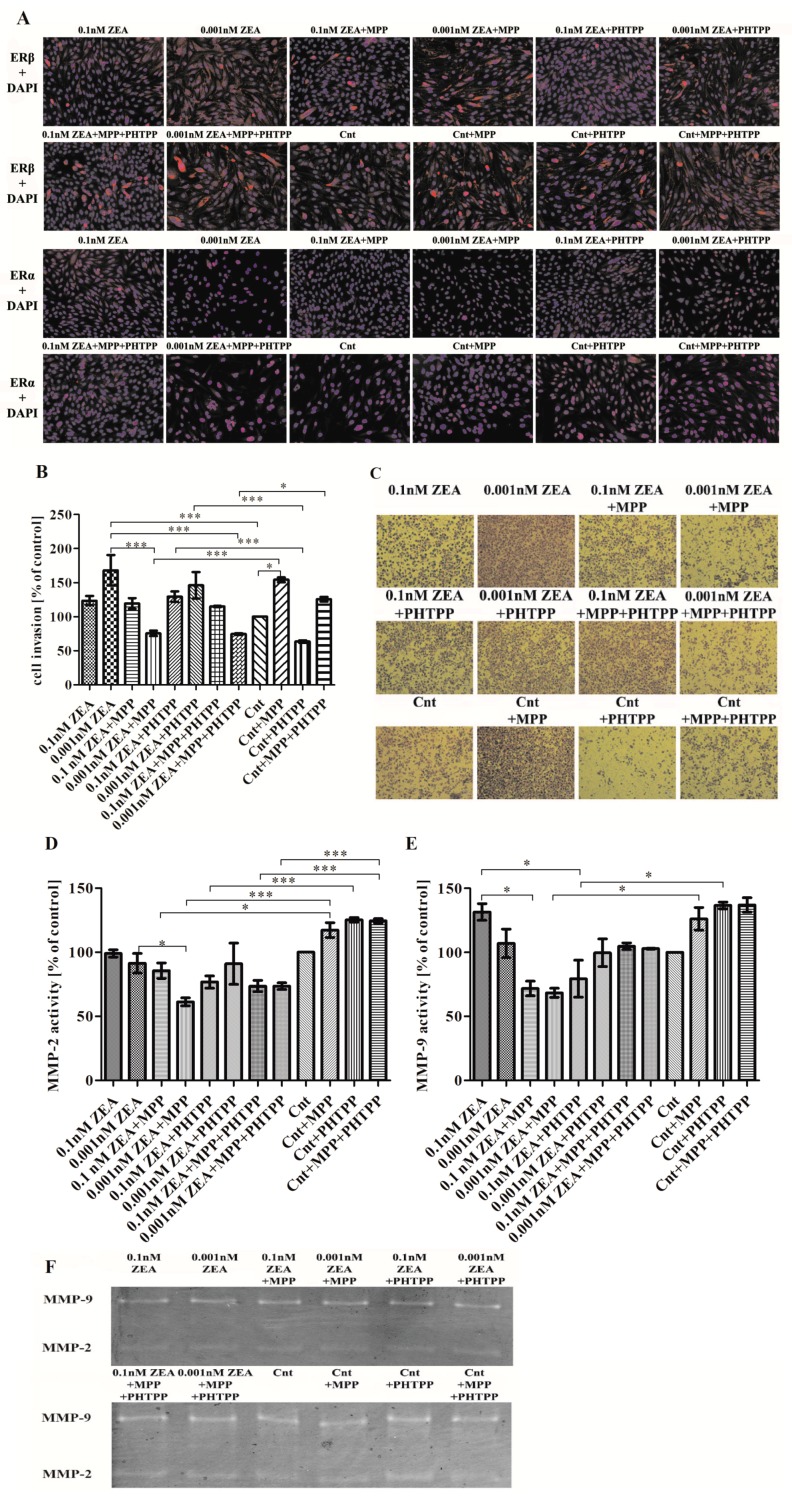

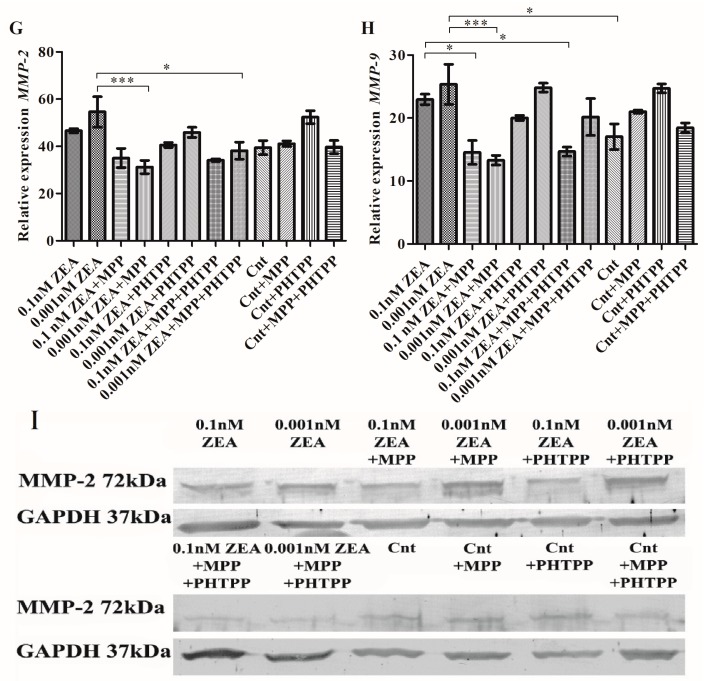
ZEA induces PC3 cell invasion dependent on ERα. (**A**) the results of ICC of ERα and ERβ (red stained) and DAPI (nuclei staining in blue); (**B**) the results from the cell invasion assay (modified Boyden chamber) are expressed as mean ± SE and presented as % of control; (**C**) representative results from cell invasion experiment, cells were stained with crystal violet and photographed in inverted microscopy; (**D**,**E**) the results from zymography assay are expressed as mean ± SE value as % of control cells; (**F**) representative results from zymography assay; (**G**,**H**) the results from the RT-qPCR study are expressed as mean ± SE and relative expression of genes was calculated as a ratio of ΔΔCt calculated expression of the gene od interest and reference genes: *H3F3A*, *RPLP0* and *RPS17*; (**I**) the results from Western blot conducted to evaluate the expression of MMP-2, GAPDH was used as a reference. Statistically significant results were marked with lines, * *p* < 0.05, *** *p* < 0.001. ICC—immunocytochemistry, ER—estrogen receptor, DAPI—4’,6-diamidino-2-phenylindole, MMP-2—metalloproteinase 2, MMP-9—metalloproteinase 9, RPLP0—60S acidic ribosomal protein P0, RPS17—40S ribosomal protein S17, H3F3A—histone H3.3, MPP—ERα antagonist, PHTPP—ERβ antagonist, ZEA—zearalenone, Cnt—control cells.

A previous study found that ZEA at concentrations of 0.1 and 0.001 nM caused an increase in PC3 cells proliferation and metabolism [12]. Our present findings indicate that while both concentrations caused an increase in cell invasion, a greater degree was observed for cells treated with 0.001 nM ZEA than control cells (*** *p* < 0.001) and non-significant for 0.1 nM ZEA (*p* > 0.05) (Figure 1B,C).

To determine whether the ERs, together or alone, influence the observed cell invasion, it was examined whether the presence of ER inhibitors has any effect on control cells. It was found that inhibition of ERα (MPP) caused a statistically significant increase of PC3 cell invasion (* *p* < 0.05) whereas inhibition of ERβ (PHTPP) had the opposite effect and was not significant (*p* > 0.05). PC3 cells treated with 0.1 nM ZEA and either MPP, PHTPP or MPP + PHTPP showed no significant change in cell invasiveness. Treatment with 0.001 nM ZEA + MPP caused a significant decrease in cell invasion compared to 0.001 nM ZEA alone (*** *p* < 0.001) or controls with MPP (*** *p* < 0.001). Treatment with 0.001 nM ZEA + PTHPP also caused a decrease but to a lesser degree and insignificantly than 0.001 nM ZEA alone (*p* > 0.05). A similar significant decrease was observed following treatment with 0.001 nM ZEA combined with MPP + PHTPP as compared to 0.001 nM ZEA alone (*** *p* < 0.001), or controls with both inhibitors (*** *p* < 0.001) (Figure 1B,C).

The next part of the study evaluated whether the observed changes in cell invasion are associated with MMP-2 and MMP-9 activity (Figure 1D,E). Treatment with 0.1 nM or 0.001 nM ZEA alone was found to have no effect on MMP-2 activity, or only slightly decreased it (*p* > 0.05) (Figure 1D). The use of 0.1 nM ZEA + MMP caused non-significant effect on treated cells, whereas significantly lower MMP-2 activity was observed following 0.001 nM ZEA + MPP treatment than in cells treated with 0.001 nM ZEA alone (* *p* < 0.05) or in control cells treated with MPP (* *p* < 0.05, *** *p* < 0.001). No significant decrease in MMP-2 activity was observed for 0.1 nM ZEA + PHTPP as well as 0.001 nM ZEA + PHTPP as compared to ZEA alone (*p* > 0.05), although a significant decrease of MMP-2 activity was observed for 0.1 nM ZEA + PHTPP as compared to controls with PHTPP (*** *p* < 0.001). For both concentrations of ZEA, treatment with both ER blockers together (MPP + PHTPP) caused MMP-2 activity to be significantly lower than in controls also treated with MPP + PHTPP (*** *p* < 0.001).

The addition of both inhibitors to controls caused an insignificant increase in MMP-9 activity compared to control cells (*p* > 0.05) (Figure 1E). The increase in MMP-9 activity caused by ZEA alone was concentration dependent and not significant as compared to controls (*p* > 0.05). Compared to treatment with ZEA alone, 0.1 nM ZEA + MMP induced significantly lower MMP-9 activity (* *p* < 0.05) than 0.001 nM ZEA + MMP (*p* > 0.05). The decrease in MMP-9 activity was significant for 0.001 nM ZEA + MPP as compared to controls with MPP (* *p* < 0.05) A statistically significant decrease in MMP-9 activity was also observed for 0.1 nM ZEA + PHTPP treatment as compared to controls with PHTPP (* *p* < 0.05). Treatment with both inhibitors had no significant effect on cell invasion at either ZEA concentration, although MMP-9 activity was reduced as compared to control (*p* > 0.05). A non-significant effect was observed on control cells after addition of MPP and PHTPP (*p* > 0.05). The representative results of zymography are presented in the Figure 1F.

The invasion of cells was also evaluated on the mRNA level (Figure 1G,H). *MMP-2* expression was upregulated after ZEA treatment and higher expression was observed for the lower concentration of ZEA (*p* > 0.05) (Figure 1G). For the control group, an increase in *MMP-2* expression was observed only following PHTPP addition and was not significant (*p* > 0.05). At lower concentration of ZEA, the addition of MPP caused a significant decrease in *MMP-2* expression compared to 0.001 nM ZEA alone (*** *p* < 0.001). Similar effect was observed for 0.1 nM ZEA + MPP dose and was non-significant as compared to ZEA alone (*p* > 0.05). The addition of PHTPP caused a similar effect but to a lower extent and non-significant (*p* > 0.05). A decrease in *MMP-2* expression was observed for ZEA + MPP + PHTPP treatment as for ZEA + MPP and this effect was significantly lower for 0.001 nM ZEA + MPP + PHTPP than 0.001 nM ZEA treatment alone (* *p* < 0.05). 

Compared to controls, *MMP-9* expression was significantly higher in cells treated with 0.001 nM ZEA (* *p* < 0.05) (Figure 1H) but insignificantly higher in cells treated with 0.1 nM ZEA (*p* > 0.05). As observed previously, the addition of inhibitors to control cells caused a decrease in *MMP-9* expression in all cases. The expression of *MMP-9* was significantly lower for 0.1 nM ZEA + MPP and 0.001 nM ZEA + MPP as compared to ZEA alone (* *p* < 0.05 and *** *p* < 0.001, respectively). Administration of 0.1 nM ZEA + PHTPP also caused a decrease in *MMP-9* expression but not a significant one; no effect was observed for 0.001 nM ZEA + PHTPP. In addition, ZEA + MPP + PHTPP treatment caused a decrease in *MMP-9* expression at both ZEA concentrations compared to ZEA treatment alone; however, this decrease was significant only for 0.1 nM ZEA (*** *p* < 0.001). 

A similar effect was observed for the Western blot results: MMP-2 expression was higher than control cells values for 0.1 nM ZEA and 0.001 nM ZEA but this decreased after the addition of MPP alone (Figure 1I). Elevated MMP-2 expression was observed after ZEA + PHTPP administration. For control cells, MMP-2 expression was suppressed by the administration of MPP or PHTPP alone and higher in both inhibitors, as compared to control cells alone. MMP-9 activity was not detectable on Western blot.

### 2.2. ZEA Modulates the Expression of EMT Genes Dependent on ERα

**Figure 2:**
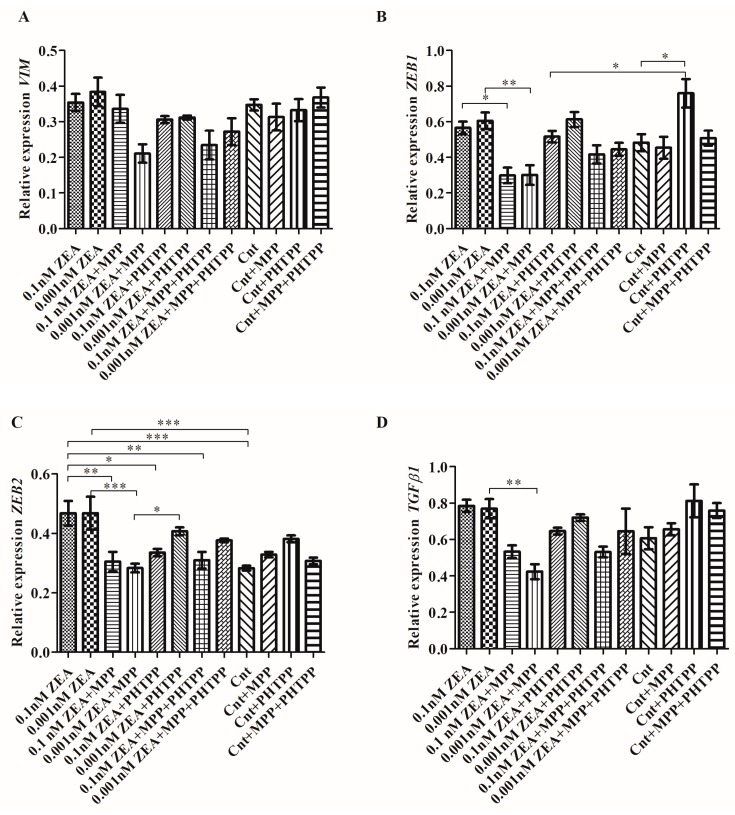
ZEA modulates expression of *VIM*, *ZEB1*, *ZEB2* and *TGFβ1* dependent on ERα expression. (**A**) the relative expression of *VIM*; (**B**) the relative expression of *ZEB1*; (**C**) the relative expression of *ZEB2*; (**D**) the relative expression of *TGFβ1*. The relative expression of genes are the results from RTqPCR calculated as the ratio of ΔΔCt calculated expression of gene of interest and reference genes *RPLP0*, *RPS17* and *H3F3A* and are expressed as mean ± SE. Statistically significant results were marked with lines, * *p* < 0.05, ** *p* < 0.01, *** *p* < 0.001. RPLP0—60S acidic ribosomal protein P0, RPS17—40S ribosomal protein S17, H3F3A—Histone H3.3, VIM—vimentin, ZEB1—Zinc finger E-box-binding homeobox 1, ZEB2—Zinc finger E-box-binding homeobox 2, TGFβ1—Transforming growth factor beta 1, MPP—ERα antagonist, PHTPP—ERβ antagonist, ZEA—zearalenone, Cnt—control.

The next part evaluated the expression of genes associated with EMT: *VIM*, *ZEB1*, *ZEB2*, *TGFβ1* (Figure 2). A tendency in increased *VIM* expression was observed for lower dose of ZEA which was decreased after blocking ERα with MPP (Figure 2A). A slight decrease was observed for PHTPP treatment and a greater decrease for MPP + PHTPP (*p* > 0.05).

A similar effect was observed for *ZEB1* and *ZEB2*. Cells treated with ZEA alone demonstrated greater *ZEB1* expression than the untreated cells (*p* > 0.05) (Figure 2B). ZEA + PHTPP treatment caused no effect on *ZEB1* expression; however, expression fell significantly following 0.1 nM ZEA + MPP or 0.001 nM ZEA + MMP treatment as compared to ZEA treatment alone (* *p* < 0.05 and ** *p* < 0.01, respectively). ZEA + MPP + PHTPP administration reduced *ZEB1* expression, although not as much as MPP alone (*p* > 0.05). Interestingly, the addition of PHTPP to untreated cells caused significantly greater *ZEB1* expression than in controls without PHTPP (* *p* < 0.05). A similar effect was observed for *ZEB2* expression, although a significant decrease was observed for treatment with MPP or PHTPP, or MPP + PHTPP as compared to ZEA alone (Figure 2C). Addition of MPP in both ZEA treatments caused significant decrease in *ZEB2* expression (** *p* < 0.01 and *** *p* < 0.001). The decrease was also observed after addition of PHTPP and MPP + PHTPP and was significant for 0.1 nM ZEA as compared to ZEA treatment alone (* *p* < 0.05 and ** *p* < 0.01, respectively). Treatment with 0.1 nM ZEA or 0.001 nM ZEA alone increased *TGFβ1* expression but this fell after treatment with ZEA + MPP; however, this decrease was only significant for 0.001 nM ZEA + MPP compared to 0.001 nM ZEA alone (** *p* < 0.01) (Figure 2D). ZEA + PHTPP caused only a slight decrease in *TGFβ1* expression and a similar decrease was observed for MPP + PHTPP (*p* > 0.05). Similar to *ZEB1*, the expression of *ZEB2* was also upregulated in control cells following treatment with PHTPP (*p* > 0.05).

### 2.3. ERs Are Crucial in ZEA-Induced PC3 Cell Migration

**Figure 3:**
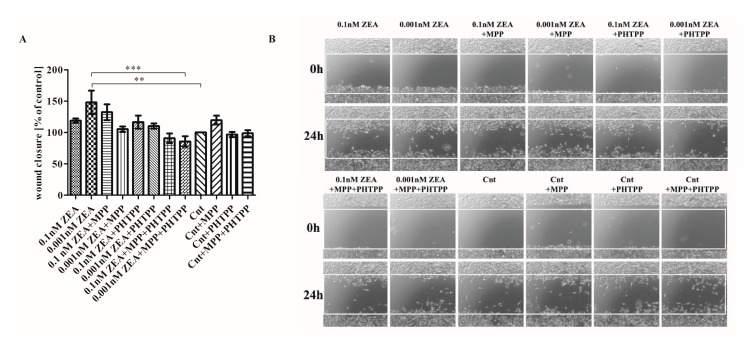
ERs are crucial in PC3 cell migration caused by ZEA. (**A**) the results of the scratch assay were expressed as a % of control cells wound closure calculated as a difference between the scratched area after 0 and 24 h, the results are expressed as mean ± SE, statistically significant differences were marked with lines, ** *p* < 0.01, *** *p* < 0.001; (**B**) representative results of the scratch assay. MPP—ERα antagonist, PHTPP—ERβ antagonist, ZEA—zearalenone, Cnt—control.

The next stage examined whether ZEA modulates the migration of PC3 cells and whether these effects are associated with ERs (Figure 3). It was found that ZEA might modulate cell migration, with those cells treated with 0.001nM ZEA demonstrating a higher level of migration than untreated cells (** *p* < 0.01) (Figure 3A). Significant decrease in cell migration was observed following the addition of MPP+PHTPP to 0.001 nM ZEA, compared to 0.01 nM ZEA alone (*** *p* < 0.001). The addition of MPP and PHTPP to 0.001 nM ZEA caused non-significant decrease in cell migration as compared to ZEA alone. For the higher ZEA concentration the migration was increased as compared to controls (*p* > 0.05); similar cell migration was observed for 0.01 nM ZEA+PHTPP but slightly lower for 0.01nM ZEA + MPP + PHTPP (*p* > 0.05). Control cells demonstrated elevated migration only following the addition of MPP (*p* > 0.05). Representative images are given in Figure 3B.

### 2.4. The Effect of ZEA on Cell Adhesion to ECM Proteins is Associated with ERα

**Figure 4:**
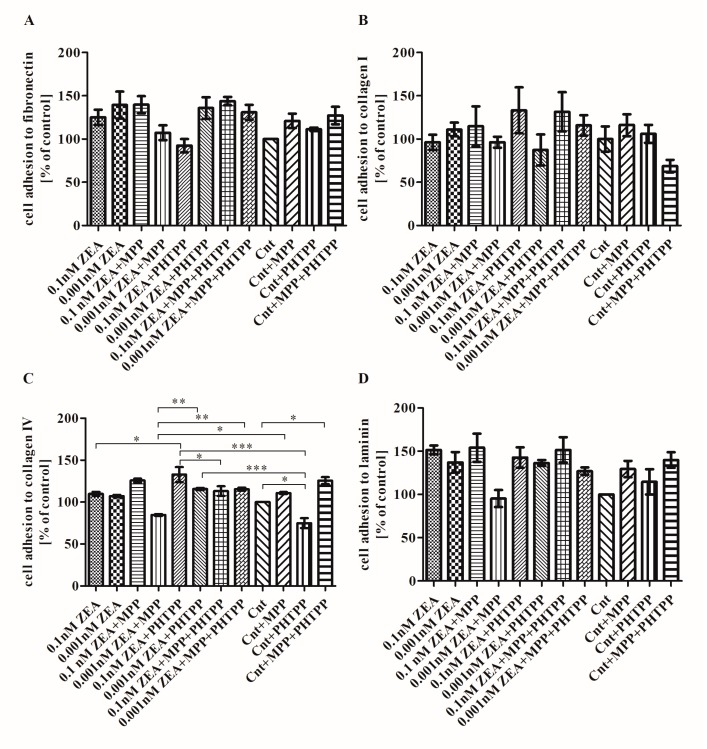
ZEA modulates the adhesion of PC3 cells to ECM proteins. (**A**) cell adhesion to fibronectin; (**B**) cell adhesion to collagen I; (**C**) cell adhesion to collagen IV; (**D**) cell adhesion to laminin. The results are expressed as mean ± SE of % of control. Statistically significant differences were marked with lines, * *p* < 0.05, ** *p* < 0.01, *** *p* < 0.001. ECM—extracellular matrix, MPP—ERα antagonist, PHTPP—ERβ antagonist, ZEA—zearalenone, Cnt—control.

The next stage examined the effect of ZEA on adhesion to the ECM proteins: collagen I, collagen IV, laminin and fibronectin. No significant changes were observed regarding PC3 cell adhesion to fibronectin, collagen I or laminin, although slightly greater adhesion was observed after treatment with ZEA (Figure 4) (*p* > 0.05). In all experiments, 0.1 nM ZEA + MPP treatment resulted in either no effect or an increase in cell adhesion, with a smaller increase observed for 0.001 nM ZEA + MPP. No such effect was observed for ZEA + PHTPP or ZEA + MPP + PHTPP, indicating that ZEA might modulate PC3 cell adhesion and ERα might be involved in this effect. Adhesion to collagen IV was only slightly elevated after ZEA treatment as compared to control cells (Figure 4C) (*p* > 0.05). Greater cell adhesion was observed for 0.1 nM ZEA + MPP but the opposite was found for 0.001 nM ZEA + MPP (*p* > 0.05). Treatment with 0.001 nM ZEA + MPP caused significantly reduced adhesion as compared to controls treated with MPP (* *p* < 0.05). Addition of PHTPP to both ZEA doses caused increase in cell adhesion as compared to ZEA alone, significant only for 0.1 nM ZEA + PHTPP (* *p* < 0.05). In both cases addition of PHTPP and MPP + PHTPP to 0.001 nM ZEA caused significant increase in cell adhesion to collagen IV as compared to 0.001 nM ZEA + MPP (** *p* < 0.01). Addition of PHTPP to both ZEA doses caused significant increase in cell adhesion as compared to controls with PHTPP (*** *p* < 0.001). Controls treated with MPP caused non-significant increase in cell adhesion to collagen IV, whereas contradictory effect was observed for addition of PHTPP as well as the similar one observed after addition of both inhibitors (* *p* < 0.05). 

## 3. Discussion

Prostate cancer is the second greatest cause of cancer- related deaths in Poland and represents a global problem in other European countries as well as the United States [18,19]. Age, ethnicity, place of residence and diet may be causative factors in prostate tumorigenesis [20]. Estrogens take part in many physiological processes in men, including both reproduction and carcinogenesis [21]. It is believed that ERα is associated with proliferation whereas ERβ plays a pro- apoptotic role [22], although some research indicates that the role of ERβ might be twofold, depending on its subtype [23].

The present study examines the proliferation-stimulating effect that low doses of ZEA (0.1 and 0.001 nM) have been found to have on PC3 cells. It examines whether these characteristics are associated with the process of cell motility and evaluates the role of estrogen receptors in this process. As ZEA has been reported to bind to both ERs in a similar way to β-estradiol, both antagonists of ERs were used. A significant finding is that our results indicate that ZEA acts directly on prostate cancer cells via ERs. A relationship was found between the increase in cell viability observed previously [12] and the increase in cell invasiveness, cell migration, MMP-2 and MMP-9 activity, as well as with the expression of genes associated with the EMT process, observed in the present study. This effect appears to be more associated with ERα than ERβ signaling, although it is possible that differences in the cell motility processes might be associated with differences in ER type.

ZEA has been reported to have concentration-dependent pro-apoptotic activity in both prostate cancer cells [12] and breast cancer cells [24]. The discrepancies in the observed results might be associated with the fact that a strong estrogenic signal is observed after treatment with the lower dose of ZEA (0.01 nM) [25], as were greater pro- proliferative and invasive effects.

It was previously reported that ZEA might modulate the proliferation and cell migration of a colon carcinoma cell line [13] as well as a breast cancer cell line [10,26]. Yip et al. showed that the observed effect of ZEA on breast cancer cells might be associated with the direct influence on ERs and the modulation of MAPK signaling pathways, which are mostly associated with ERα activation [26]. Our results also confirm that the pro-invasive effect of ZEA is associated with the activation of ERα receptor rather than ERβ. In breast cancer cells, ERα is responsible for the proliferation and survival of cells due its two-fold action: via classical ERs activation and gene regulation through estrogen response elements (ERE), as well as via non-classical activation through signal transduction via Ras/mitogen-activated protein kinase (MAPK) and phosphoinositide 3-kinase (PI3K/Akt) [27].

ZEA is known to be a selective modulator of estrogen receptors (SERM) and an endocrine disruptor chemical (EDC) because of its hormonal-balance disturbances in animals and humans [3]. This has been confirmed by other studies where ZEA was reported to modulate the proliferation of cells through ERs [28,29]. Similar to estradiol, ZEA might modulate the process of both EMT and cell invasiveness. Estrogens have been reported to modulate cell invasion via MMP-2 up-regulation [30]. Our results show that ZEA might up-regulate the expression of both MMP-2 and MMP-9; however, the blockage of ERα with MPP diminished both their activity and expression. Moreover, those changes were also confirmed by up-regulation of *TGFβ1*, a multifunctional cytokine known to modulate the expression of MMP, as well as other ECM components [30]. Interestingly, we observed that changes in the adhesion to ECM molecules after ZEA treatment were not so spectacular and a visible decrease in adhesion was observed only for the lower dose of ZEA. This fact might be associated with previous observations indicating that 0.001 nM ZEA had a greater stimulatory effect on cell proliferation than 0.1 nM ZEA [13].

EMT is a complex process mainly associated with embryogenesis and development; in addition to basic physiological process, it is also associated with cancer progression and metastasis [17]. Recent research indicates that ERs might also be involved in EMT in prostate tissue, mainly by modulation of MMP-2, MMP-3 [31] via ERα, as well as VEGF and HIF-1α via ERβ activation [32]. Our present findings indicate that ZEA is able to modulate MMP-2 and MMP-9 activity and expression, as well as the expression of other EMT genes: *VIM*, *ZEB1*, *ZEB2* and *TGFβ1*. Moreover, our results suggest that ERα is more closely associated with observed effect of ZEA than ERβ, due to the decrease in expression observed after the addition of MPP, an ERα antagonist.

Our findings confirm that ZEA might act as estrogen in prostate tissue and that it modulates both hormonal balance and tumorigenesis [3]. The estrogenic activity of ZEA in prostate tissue, possibly acting through ERα, whose effect might be associated with the proliferation, invasiveness and migration of cancer cells, is similar to those that caused by estrogen: It is known that generally ERα is responsible for the proliferation of prostate cancer cells, whereas ERβ is believed to have the opposite effect [32,33]. Moreover, up-regulation of the *TGFβ1* gene, as well as *ZEB1*, indicates that ZEA-induced proliferation might be associated with MAPK signaling, known to be involved with ERα activity in prostate tissue [34], although this statement needs to be confirmed in further studies.

## 4. Conclusions

To our knowledge, this is the first study to demonstrate that the proliferative effect of ZEA on prostate cancer cells is associated with increased cell invasiveness and migration and that these processes are probably triggered by ERα. Due to the fact that more studies have been carried out to present the dual role of ZEA on cancer cells, a possible explanation of these effects is associated with the present and direct stimulation of one type of ER, i.e., ERα or ERβ, which are known to play complex and contradictory roles in the process of carcinogenesis. To confirm this statement, more research studies, including in vivo models should be carried out to evaluate the effect of ZEA contamination in the diet on the process of carcinogenesis in hormone-sensitive tissues.

## 5. Materials and Methods

### 5.1. Cell Culture

The PC3 human prostate cancer cell line was purchased from DSMZ (Leibniz Institute DSMZ- German Collection of Microorganisms and Cell Cultures, Braunschweig, Germany) cells were cultured in a humidified incubator at 37 °C with 5% CO_2_ in RPMI 1640 medium (Thermo Fisher Scientific Inc/Life technologies, Waltham, MA, USA) supplemented with 10% heat-inactivated Fetal Bovine Serum (FBS) (Thermo Fisher Scientific Inc/Life technologies, Waltham, MA, USA), 1 mM Sodium Pyruvate (Thermo Fisher Scientific Inc/Life technologies, Waltham, MA, USA), 10 mM HEPES Buffer (Thermo Fisher Scientific Inc/Life technologies, Waltham, MA, USA) and antibiotics (Penicillin 50 U/mL; Streptomycin 50 μg/mL; Neomycin 100 μg/mL) (Thermo Fisher Scientific Inc/Life technologies, Waltham, MA, USA). During each experiment, PC3 cells were serum deprived and cultured in phenol red-free medium.

Stock solutions of ZEA (Sigma-Aldrich, Saint Louis, MO, USA). MPP (Santa Cruz Biotechnology, Dallas, TX, USA) and PHTPP (Santa Cruz Biotechnology, Dallas, TX, USA) were prepared in methanol, water and DMSO, respectively. The final concentrations of tested ZEA, MPP and PHTPP were achieved by the addition of phenol red- free RPMI culture medium. To exclude the potential influence of the solvent, the cells were treated with methanol/DMSO at the highest concentrations used in the study and no statistically significant decrease in viability was observed. Therefore, for the rest of experiment, non-treated cells were used as control cells. For all experiments, the cells were treated with ZEA (0.1 nM, 0.001 nM), combinations of ZEA with 100 pM MPP and/or 100 pM PHTPP (0.1 nM ZEA + MPP, 0.001 nM ZEA + MPP, 0.1 nM ZEA + PHTPP, 0.001 nM ZEA + PHTPP, 0.1 nM ZEA + MPP + PHTPP, 0.001 nM ZEA + MPP + PHTPP) or inhibitors alone (Cnt + MPP, Cnt + PHTPP, Cnt + MPP +PHTPP) to evaluate its effect on control cells. The cells were incubated for 24 or 72 h as indicated.

### 5.2. Immunocytochemistry (ICC)

To prepare the cultures, 15 × 10^4^ cells were seeded on a 96-well plate and incubated under standard conditions. Next day, the medium was exchanged for the experimental medium and the cells were incubated for 72 h. After the incubation time, the media were removed and wells were fixed with 4% paraformaldehyde (PFA), washed three times with phosphate- buffered saline (PBS) and permabilizated in PBST (PBS-Tween 20) for 10 min. The wells were then blocked in 1% non- fat milk in PBST for 30 min and incubated overnight at 4 °C with primary antibodies against ERα (1:50, sc-8005, Santa Cruz Biotechnology, Dallas, TX, USA), ERβ (1:50, sc-6820, Santa Cruz Biotechnology, Dallas, TX, USA) in PBST. The following day, the plate was washed three times with PBS and incubated with secondary antibodies: AlexaFluor^®^ 594 rabbit anti-goat IgG and AlexaFluor^®^ 594 goat anti-mouse IgG (1:100) in PBST for one hour. Following this, they were washed once again with PBS and stained with DAPI (Sigma Aldrich, Saint Louis, MO, USA) before being washed two times with PBS and visualized at a FLoid Cell Imaging Station (ThermoFisher Scientific, Waltham, MA, USA).

### 5.3. Cell Adhesion Assay

The cells were seeded on six-well plates at a density of 5 × 10^5^/well and incubated to reach 80% confluence (one to two days). The medium was then exchanged for experimental media. After 72 h, the cells were detached with Trypsin/EDTA solution (ThermoFisher Scientific Inc., Waltham, MA, USA) and counted on an automated Cell Counter (ThermoFisher Scientific Inc, Waltham, MA, USA). Following this, 1 × 10^5^ of cells were seeded on 24-well plates coated with collagen type I, collagen type IV, laminin or fibronectin (Corning, New York, NY, USA). The plates were then incubated at 37 °C in a standard culture conditions for 1.5 h. After the incubation medium was removed, the wells were washed three times with PBS to remove the unattached cells. The cells were then stained with 0.1% crystal violet for 10 min, washed three times with water and dried. The cells were dissolved in 10% acetic acid. The solute mixture was transferred to a 96-well plate (200 µL/well) and absorbance was measured at 550 nm on an ELX U808IU plate reader (BioTek, Winooski, VT, USA). The experiment was conducted in three replicates.

### 5.4. Monolayer Wound Migration Assay

PC3 cells were seeded on 12-well plates and left to reach 100% confluence. Cell monolayers were wounded as described previously [35] and the medium was exchanged for experimental medium supplemented with 0.5 mM solution of hydroxyurea (Sigma-Aldrich, Saint Louis, MO, USA). At the beginning of incubation time (0 h) and after 24 h, cells were photographed with an Olympus DP20 camera (Olympus, Shinjuku, Tokyo, Japan), magnitude 40×. Migration was calculated as the difference between the area of the wound after 24 h and 0 h and expressed as % of control (migration assay for control cells). All calculations were performed in MicroImage (Olympus, Shinjuku, Tokyo, Japan). The experiment was conducted in triplicate.

### 5.5. Cell Invasion Assay

Units of 1.5 × 10^5^ of cells in 600 µL of experimental media were seeded on 8 μm pore transwell 12-well inserts coated with 100 μL Geltrex^TM^ (ThermoFisher Scientific Inc, Waltham, MA, USA) at a final concentration of 200 μg/mL (modified Boyden chamber assay) and incubated for 24 h on companion plates filled with 2 mL of standard culture medium (10% FBS) without antibiotics. The cell inserts and companion plates were washed three times with PBS, fixed with 4% PFA (five minutes), washed once again with PBS and stained with 0.1% crystal violet for 10 min. The non-invasive cells that remained on the upper surface of the filter were removed with cotton wool. The stained invasive cells on the inserts were photographed with an Olympus DP20 camera before being dissolved in 200 µL of 10% acetic acid and transferred to a 96-well plate. The absorbance was measured at 550 nm on an ELX U808IU plate reader. The experiment was conducted in triplicate.

### 5.6. Gelatin Zymography

Units of 5 × 10^5^ cells were seeded onto six-well plates and incubated until 90% confluence. Next, the cells were treated with experimental media, as described previously, for 24 h, after which time, the media were collected. The concentration of the protein content was calculated with a QubitR Protein Assay Kit (ThermoFisher Scientific, Inc, Waltham, MA, USA) according to the manufacturer’s instructions. After assay, 7 µg of protein was loaded on 10% gelatin zymography gels and subjected to electrophoresis (120 V, two hours, on ice) and then incubated in 2.5% Triton X-100 (Sigma-Aldrich, Saint Louis, MO, USA) two times for 30 min. Next gels were incubated 48 h in developing buffer in 37 °C to enable the determination of total proteolytic MMP activity. The next day, the gels were stained with Coomassie brilliant blue (Sigma-Aldrich, Saint Louis, MO, USA) and destained with 50% methanol and 20% acetic acid. Areas of enzymatic activity appeared as clear bands over a dark blue background. Gels were scanned and the intensity of bands was calculated in inverted negative image using ImageJ software (Wayne Rasband, National Institutes of Health, Bethesda, MD, USA). The experiment was run in triplicate.

### 5.7. Western Blot

The cells were seeded on Petri dishes and induced as described previously. After 72 h, the cells were detached and the protein was isolated with RIPA buffer (Sigma-Aldrich, Saint Louis, MO, USA) as described previously [12]. Membranes were blocked in 5% fat-free milk in TBST buffer prior to overnight incubation in 4 °C in the primary antibodies anti- MMP-2 (1:200 in 1% fat-free milk, SantaCruz Biotechnology, Dallas, TX, USA) or anti-GAPDH (1:1000, SantaCruz Biotechnology, Dallas, TX, USA) as a reference. After incubation, the membranes were washed three times with TBST buffer and incubated with anti-rabbit and anti-mouse secondary antibodies (1:15,000, Sigma-Aldrich, Saint Louis, MO, USA) for MMP-2 and GAPDH, respectively, for four hours at 4 °C. The membranes were washed once again and the bands were visualized with using Novex^®^ AP Chromogenic Substrate (BCIP/NBT) (Thermo Fisher Scientific, Inc, Waltham, MA, USA). Densitometric analysis was conducted with ImageJ [36] (National Institutes of Health, Bethesda, MD, USA). The experiment was conducted in triplicate.

### 5.8. Real Time qPCR (RTqPCR)

cDNA was synthesized from 5 μg of total RNA using ImProm RT-IITM reverse transcriptase (Promega, Madison, WI, USA) according to the manufacturer’s instructions. Reverse transcription was conducted as described previously [17]. A LightCycler 96 (Roche) was used to perform the RT-qPCR reaction with 2 μL of cDNA. Primers were designed using Primer3 software [37] (Cambridge, MA, USA) (Table 1). The analysis was performed using DFS-Taq DNA Polymerase kit (BIORON, Ludwigshafen, Germany) according to the manufacturer’s instructions. The Human Reference RNA (Stratagene, San Diego, CA, USA) was used as a calibrator for each reaction. The relative expressions of *TGFβ1*, *VIM*, *ZEB1*, *ZEB2*, *MMP-2* and *MMP-9* were normalized to three reference genes: ribosomal protein S17 (*RPS17*), ribosomal protein P0 (*RPLP0*) and histone H3.3A (*H3F3A*). In order to avoid detection of non-specific products for each reaction, melting curve analysis was performed. Meting curve analysis showed specificity of the product for each primer set. The efficacy of all primers sets determined on standard curve was from 91.72 to 121.82%. The linearity of each primer set was determined (R^2^) in range 0.93–0.99. The qPCR array data was analyzed using the ΔΔCt method. The results were obtained in duplicate from three repeats of the experiment.

### 5.9. Statistical Analysis

The results are expressed as a mean ± SE and were analyzed with one-way ANOVA. Values below *p* = 0.05 were considered statistically significant. GraphPad Prism (GraphPad Software, La Jolla, CA, USA) was used for all statistical analyses.

## Figures and Tables

**Table 1:** Primers used in RTqPCR. *VIM*-vimentin; *ZEB1*—zinc finger E-box binding homolog 1; *TGFβ1*—transforming growth factor β1; *ESR1*-estrogen receptor α; *MMP-2*—metalloproteinase 2; *MMP-9*—metalloproteinase 9; *RPLP0*—ribosomal protein P0; *RPS17*—ribosomal protein R17; *H3F3A*—histone H3.3A; bp—base pair.

Gene	Primer Sequence (5’-3’)	Product Size (bp)
*H3F3A*	AGGACTTTAAAAGATCTGCGCTTCCAGAG ACCAGATAGGCCTCACTTGCCTCCTGC	74
*MMP-2*	ACCAGCTGGCCTAGTGATGATGTT TGTCCTTCAGCACAAACAGGTTGC	184
*MMP-9*	CTGGCAGGGTTTCCCATCAG GCAGTACCACGGCCAACTAC	101
*RPLP0*	ACGGATTACACCTTCCCACTTGCTAAAAGGTC AGCCACAAAGGCAGATGGATCAGCCAAG	69
*RPS17*	AAGCGCGTGTGCGAGGAGATCG TCGCTTCATCAGATGCGTGACATAACCTG	87
*TGFβ1*	CAATTCCTGGCGATACCTCAG GCACAACTCCGGTGACATCAA	86
*ZEB1*	GGAAATCAGGATGAAAGACA CACACAAATCACAAGCATAC	136
*ZEB2*	CTAACCCAAGGAGCAGGTAATC GTGAATTCGCAGGTGTTCTTTC	96
*VIM*	AGCCGAAAACACCCTGCAAT CGTTCAAGGTCAAGACGTC	72
